# Moderately Hypofractionated Radiotherapy Alone for Stage I-IIB Non-small Cell Lung Cancer

**DOI:** 10.7759/cureus.4969

**Published:** 2019-06-22

**Authors:** Ryan T Hughes, Corbin A Helis, Michael H Soike, Beverly J Levine, Michael Farris, Arthur W Blackstock

**Affiliations:** 1 Radiation Oncology, Wake Forest School of Medicine, Winston-Salem, USA; 2 Epidemiology, Wake Forest School of Medicine, Winston-Salem, USA

**Keywords:** hypofractionated radiation therapy, non-small cell lung cancer, ultracentral, medically inoperable, surgically inoperable

## Abstract

Introduction: The optimal management of patients with early non-small cell lung cancer (NSCLC) not amenable to surgical resection or stereotactic body radiotherapy (SBRT) or those with hilar nodal involvement ineligible for surgery or concurrent chemoradiotherapy is unclear. This report describes survival outcomes and toxicity profiles of patients treated with hypofractionated radiotherapy (HRT) alone.

Methods: A total of 52 patients with Stage I-IIB NSCLC treated with HRT alone between 2010-2018 were reviewed. Patients were categorized as having ultracentral tumors if the planning target volume contacted or overlapped the proximal bronchial tree, esophagus, pulmonary vein or artery. Overall survival (OS) and progression-free survival (PFS) were estimated using the Kaplan-Meier method and the competing risk cumulative incidence of locoregional failure (LRF) and distant failure (DF) were estimated using death without failure as a competing risk. Pneumonitis and esophagitis rates were evaluated as per Acute Common Terminology Criteria for Adverse Events (CTCAE) version 4.0.

Results: Of the 52 patients analyzed, 50 patients were treated with radiotherapy alone to a dose of 70.2 Gy in 26 fractions, one patient was treated with 68 Gy in 25 fractions and one patient was treated with 65 Gy in 26 fractions. The median age was 72 (range 48-89), 42% of patients had an Eastern Cooperative Oncology Group (ECOG) performance status of 2-3, 46% were stage I and 54% were stage II. Hilar nodal involvement was present in 21% of patients and 74% of node-negative patients had ultracentral primary tumors. Median OS was 39.6 months and the median PFS was 21.0 months. Overall three-year cumulative incidence of LRF and DF were 32% and 34%, respectively. Grade 3 pneumonitis occurred in two (4%) patients. No grade 3+ acute esophagitis or grade 4-5 toxicities were observed.

Conclusion: Hypofractionated thoracic radiotherapy consisting of 70.2 Gy is well-tolerated and results in favorable locoregional control for stage I-IIB patients who are not candidates for SBRT, surgery, or concurrent chemoradiotherapy.

## Introduction

Patients with operable, early stage non-small cell lung cancer (NSCLC) may be appropriately managed with either surgical resection or stereotactic body radiotherapy (SBRT). For inoperable patients or those who decline surgery, SBRT offers excellent local control and limited toxicity [1-2]. However, patients with tumors larger than 5 cm, those within 2 cm of the proximal bronchial tree or overlapping mediastinal structures (ultracentral) are at increased risk for treatment-related morbidity after SBRT if normal tissue constraints cannot be achieved [3-5]. Various hypofractionated radiotherapy (HRT) regimens have been reported for the management of these patients [6-8], including the Cancer and Leukemia Group B (CALGB) 39904 study [9]. This phase I study evaluated various HRT regimens to a total dose of 70 Gy in 17-29 fractions without concurrent chemotherapy for early stage T1-T2N0 NSCLC. Disease control was favorable and no dose-limiting toxicity was observed.

The non-surgical management of these patients with stage I-IIB NSCLC not amenable to SBRT can be challenging. Various definitive radiotherapeutic regimens have been reported, though no clear standard exists [10]. Within the SBRT literature, dose-escalation to an approximate biologically effective dose (BED)10 of 100 Gy may improve local control [11-12]. Emerging data in the context of locally advanced NSCLC provide a rationale for dose-escalation using altered fractionation with or without concurrent or sequential chemotherapy [13-14]. The BED10 of these regimens ranges from 58.5-78 Gy.

Current guidelines recommend concurrent chemoradiotherapy followed by consolidative immunotherapy for patients with stage II NSCLC with hilar involvement [15]. However, various comorbidities, medical contraindications or patient preference sometimes preclude the delivery of concurrent chemotherapy. In these cases, radiotherapy alone is often utilized. Multiple studies have employed various altered fractionation regimens in the management of NSCLC patients with radiotherapy alone and a benefit to survival outcomes has been suggested with the use of modified fractionation compared to conventional fractionation [16]. In patients with medically inoperable disease that are not candidates for chemotherapy, 45 Gy delivered in 15 fractions (BED10 = 58.5 Gy) is a commonly utilized HRT schedule with a favorable toxicity profile and locoregional tumor control rates between 58% and 70%, similar to that of conventionally-fractionated RT alone [17-18]. However, stage II patients were not well-represented in these two studies which also reported distant failure in approximately 40%-46%.

At our institution, patients with stage I-IIB NSCLC who were ineligible for either resection, SBRT or combined chemoradiotherapy were treated with moderately hypofractionated radiotherapy alone based on CALGB 39904. The most common HRT regimen utilized was 70.2 Gy in 26 fractions (BED10 = 89.15 Gy) [19]. We aimed to evaluate the tumor control, survival outcomes, and toxicity profile of this regimen.

## Materials and methods

Patient population, evaluation, and treatment

In an Institutional Review Board-approved retrospective analysis, we reviewed the records of 92 patients with NSCLC receiving curative-intent HRT at the Wake Forest University Comprehensive Cancer Center between January of 2010 and June of 2018. Study data were collected and managed using research electronic data capture (REDCap) tools hosted at the Wake Forest School of Medicine Clinical and Translational Science Institute [20]. We identified 52 patients with newly-diagnosed American Joint Committee on Cancer 7th Edition Stage I-IIB NSCLC available for analysis. All patients underwent multidisciplinary thoracic oncology evaluation and all underwent staging imaging using [18F]fluorodeoxyglucose positron emission tomography. Brain magnetic resonance imaging (MRI) was employed in 13 of 24 stage I patients and 15 of 28 stage II patients. Patients were thoroughly counseled regarding the potential treatment options including surgery, radiotherapy, chemotherapy, or a combination. Those who were surgical candidates but declined surgery were offered radiotherapy. Treatment volumes were generated using four-dimensional computed tomography (CT) simulation as previously described [19]. The dose regimen prescribed was determined at the discretion of the treating physician when considering the target and normal tissues at risk on an individual basis. Per institutional preference, the majority of patients (96%) were treated with 70.2 Gy in 26 fractions using 3D conformal radiotherapy (3D-CRT), intensity-modulated radiotherapy (IMRT) or volumetric modulated arc therapy (VMAT). Elective nodal irradiation was not performed. Post-treatment systemic therapy was not routinely utilized.

Available treatment plans were reviewed to define the location of the primary tumor, its proximity to central structures, as well as dosimetric factors. The primary tumor size was defined by its greatest diameter (in cm) in any dimension. Dosimetric indices that were collected included the volume of the planning target volume (PTV), percentage of bilateral lung receiving 5, 20, 30 and 40 Gy (V5, V20, V30, and V40) and esophagus maximum dose were abstracted from the radiotherapy treatment planning system. Given the retrospective nature of this study and the inconsistency of documentation of cardiac outcomes, heart dosimetric parameters were not collected. Patients were categorized as having ultracentral tumors if the PTV contacted or overlapped the proximal bronchial tree, esophagus, pulmonary vein or artery, as previously defined [8]. Due to the inconsistency in the definition of ultracentral lung tumors, we chose not to deviate from this definition which does not include the heart or pericardium [5,8]. The proximal bronchial tree was defined according to the Radiation Therapy Oncology Group (RTOG) guidelines as the central airway from the distal trachea to the branching of the lobar bronchi [21].

Outcomes

Outcome measures were estimated from the date of completion of radiotherapy. Local failure (LF) was defined as either biopsy-proven disease or any radiographic evidence of recurrence at the treated primary site after completion of therapy that was recognized as such within the medical record. Regional failure was defined as the development of pathologic or radiographic evidence of disease in the regional lymph node stations. Locoregional failure (LRF) represents the occurrence of either local failure, regional failure, or both. Distant failure (DF) was defined as any pathologic or radiographic evidence of distant metastases. Overall survival was defined as the duration of time from completion of HRT to death from any cause or last follow-up. Progression-free survival was defined as the duration of time from completion of HRT to any progression event (local, regional, and/or distant failure), death from any cause, or last follow-up. Lung and esophageal toxicity were graded per the Common Terminology Criteria for Adverse Events (CTCAE) version 4.0 based upon review of the medical record [22]. Other toxicity data collected included objective measures such as steroid use for respiratory symptoms within 90 days (whether or not this was attributed to pneumonitis versus an unrelated indication) and any esophageal toxicity necessitating dilation, instrumentation, or feeding tube support.

Statistical analysis

Data were summarized using count (frequency) for categorical and median (range) for continuous variables. Time to event analyses were performed from the date of completion of radiotherapy. Duration of follow-up was estimated using the reverse Kaplan-Meier method. OS and PFS were estimated using the Kaplan-Meier method and compared using the log-rank test. Cumulative incidence (CI) of LF, RF, DF, and LRF were estimated using competing risk methodology with death without failure as the competing risk and compared using Gray’s test. Lung and esophageal dosimetric parameters were evaluated for association with pneumonitis and esophagitis using bivariate logistic regression and the Mann-Whitney U test. Cases with missing data for a given variable were excluded from analysis. All statistical analyses were performed using R version 3.5 (R Foundation for Statistical Computing, Vienna, Austria).

## Results

Study population

Patient and disease characteristics are described in Table 1. The majority of patients (58%) had good performance status (Eastern Cooperative Oncology Group (ECOG) 0-1) and most were former smokers (82%). T1-2 tumors were most common (83%) and 21% had N1 disease. Of 52 patients, 39 (75%) had either ultracentral primary tumors or hilar lymph node involvement. Reasons the patients did not undergo surgery included the following: surgically unresectable disease, medically inoperable patient, both, or patient declined surgery.

**Table 1:** Patient and Disease Characteristics cm, centimeter; ECOG, Eastern Cooperative Oncology Group; n, number; NSCLC, non-small cell lung cancer; NOS, not otherwise specified. ^a^Patient with N1 nodal involvement without evidence of a primary tumor. ^b^PTV contacts proximal bronchial tree, trachea, esophagus, pulmonary vein, or pulmonary artery among 38 node-negative patients with evaluable radiotherapy treatment plans.

	Value (n=52)
Age at Diagnosis (years), median (range)	72 (48-89)
ECOG Performance Status, n (%)	
0	4 (8)
1	26 (50)
2	14 (27)
3	8 (15)
Histology, n (%)	
Adenocarcinoma	16 (31)
Squamous cell carcinoma	26 (50)
NSCLC NOS	6 (12)
Not biopsied	4 (8)
Smoking Status, n (%)	
Current smoker	9 (18)
Former smoker	42 (82)
Smoking History (pack-years), median (range)	50 (4-260)
Tumor Stage, n (%)	
T0^a^	1 (2)
T1	17 (33)
T2	26 (50)
T3	8 (15)
Nodal Stage, n (%)	
N0	41 (79)
N1	11 (21)
Clinical Stage, n (%)	
IA	14 (27)
IB	10 (19)
IIA	17 (33)
IIB	11 (21)
Ultracentral^b^, n (%)	
Yes	28 (74)
No	10 (26)
Primary Tumor Size (cm), median (range)	3.1 (0.9-7.4)
Primary Tumor ≥5 cm, n (%)	11 (21)
Baseline Oxygen Dependence, n (%)	10 (20)
Surgical Candidacy, n (%)	
Unresectable	8 (15)
Medically Inoperable	30 (58)
Both Unresectable and Medically Inoperable	3 (6)
Declined Surgery	11 (21)

The most commonly utilized fractionation was 70.2 Gy in 26 fractions (50 of 52) and the majority of patients (90%) were treated using 3D-CRT (Table 2). No patients received chemotherapy or immunotherapy after irradiation. Median follow-up was 43.3 months (95% CI 30.9-NC).

**Table 2:** Treatment Characteristics 3D-CRT, three-dimensional conformal radiotherapy; cm, centimeter; HRT, hypofractionated radiotherapy; IMRT, intensity-modulated radiotherapy; Gy, Gray; n, number; PTV, planning target volume; VMAT, volumetric modulated arc therapy *Proximal bronchial tree, esophagus, heart/great vessels.

	Value (n=52)
Reason for HRT, n (%)	
Proximity to critical structures*	26 (50)
Ineligible for other therapies	12 (23
Size of target	14 (27)
Dose / Number of Fractions, n (%)	
70 Gy / 26	50 (96)
68 Gy / 25	1 (2)
65 Gy / 26	1 (2)
Modality, n (%)	
3D-CRT	47 (90)
IMRT/VMAT	5 (10)
PTV Volume (cm^3^), median (range)	103.3 (14.2-594.7)
Percent bilateral lung receiving specified dose, median (range)	
5 Gy	45 (16-75)
20 Gy	20 (4-35)
30 Gy	15 (2-29)
40 Gy	10 (1-25)
Mean Lung Dose (Gy), median (range)	12.07 (3.38-20.52)
Esophagus maximum dose (Gy), median (range)	32.4 (4.7-75.1)

Survival and disease control

**Figure 1:**
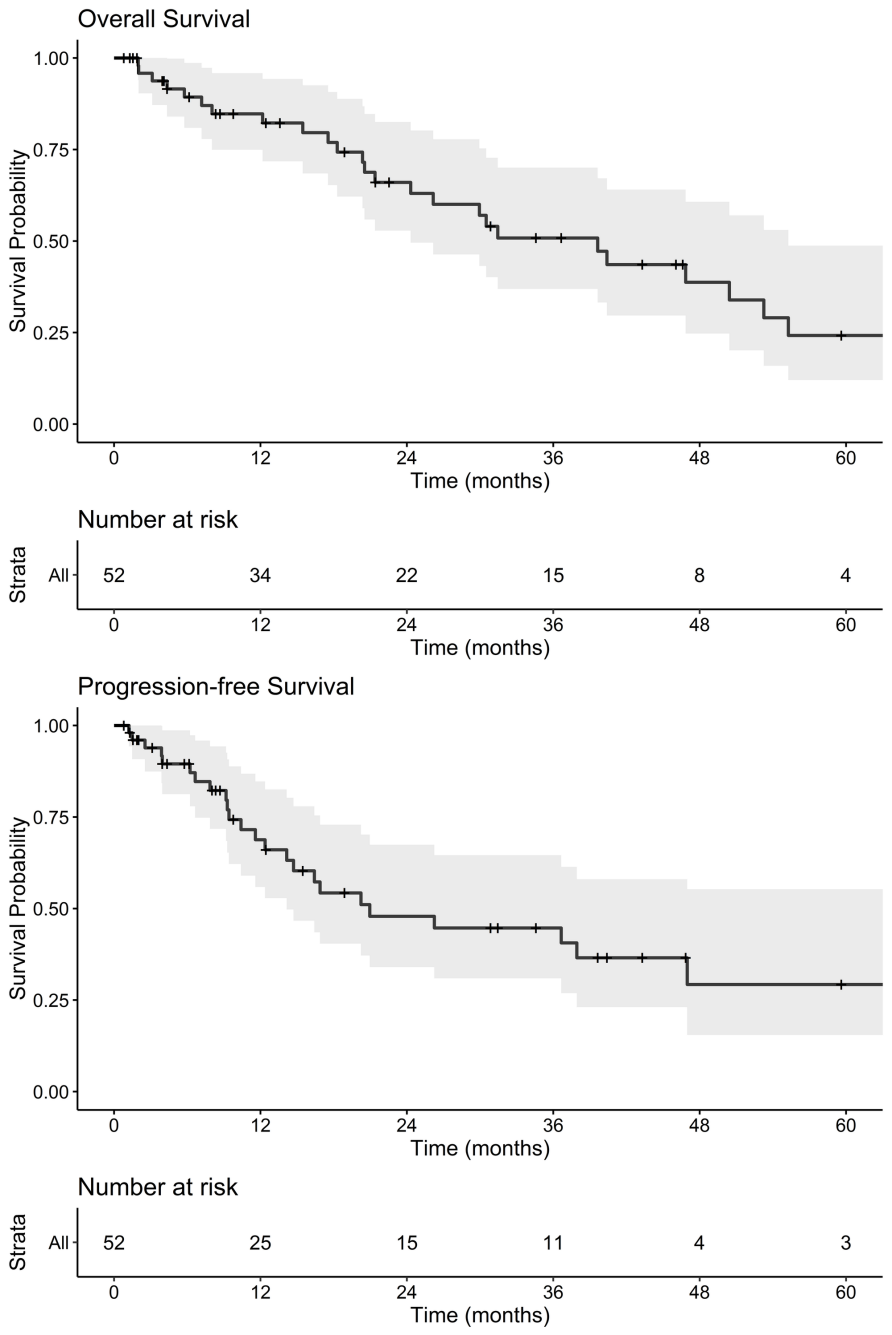
Kaplan-Meier Plots of Overall Survival and Progression-free Survival

**Figure 2:**
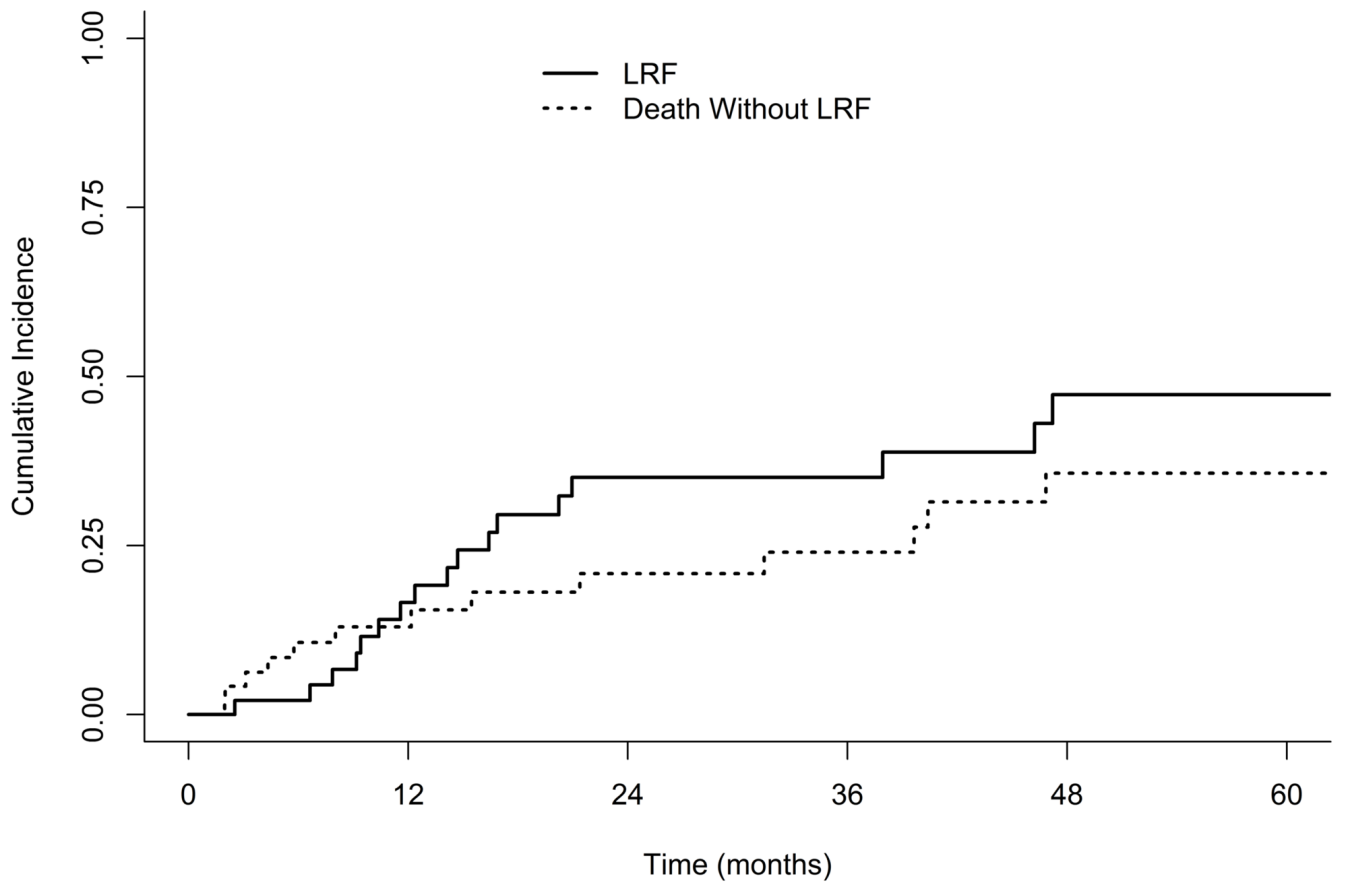
Competing-risk Cumulative Incidence of Locoregional Failure LRF, locoregional failure.

**Figure 3:**
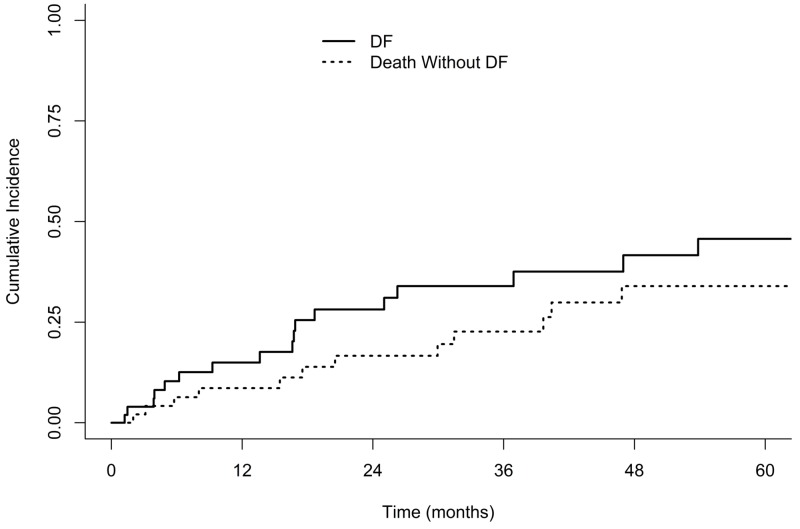
Competing-risk Cumulative Incidence of Distant Failure DF, distant failure.

Median OS and PFS for the entire cohort were 39.6 months (95% confidence interval [CI], 24.3-55.3) and 21.0 months (95% CI 14.1-not calculated [NC]), respectively (Figure 1). Median OS for node-positive versus node-negative patients was 24.3 months vs. 39.6 months (log-rank p=0.65) and the median PFS was 16.4 vs. 21.0 months (p=0.43), respectively. One- and three-year PFS was 75% and 45% for stage I and 63% and 45% for stage II patients. Cumulative incidence of LRF in the entire cohort at 3 years was 35% (Figure 2). Three-year cumulative incidence of locoregional failure for stage I and II patients was 25% and 45%, respectively (Gray’s p=0.20). Incidence of distant failure at three years was 34% overall, as seen in Figure 3; the three-year cumulative incidence of distant failure was 45% in patients with stage I and 22% in those with stage II disease (p=0.30). The most common site of distant failure in stage I patients was the lung parenchyma (three patients), followed by the brain (two), liver (two), pleural effusion (two), bone (one), abdominal organs (one) and other (one). A similar pattern was observed for stage II patients, with distant failure occurring in the lung parenchyma in four patients, pleural effusion in two, brain in one, bone in one, and other in three. Node-positive patients, compared to node-negative patients, had similar three-year CI of locoregional failure (41% v. 33%, p=0.48) and distant failure (33% vs. 35%, p=0.95). Three-year cumulative incidence of local, regional and distant failure were as follows: 25%, 10%, and 45% in stage I patients and 19%, 35%, and 22% in stage II patients.

Treatment-related toxicity

Two (4%) patients developed grade 3 pneumonitis and 9 (17%) patients required a course of steroids for respiratory symptoms. Of the two patients with Grade 3 pneumonitis, both had ultracentral primary tumors. In these patients, the V20 of the bilateral lungs were 33% and 15%. No correlation was observed between the grade of pneumonitis and bilateral lung V5, V20, V30, V40 or mean lung dose (Table 3). Bivariate logistic regression models did not identify a lung dose parameter associated with clinically significant pneumonitis (grade ≥2). No patients with baseline oxygen dependence experienced grade 2+ pneumonitis, nor did those five patients treated with intensity-modulated radiotherapy (IMRT) or volumetric modulated arc therapy (VMAT). The maximum dose to the esophagus was available in six of the eight patients experiencing acute grade 2 esophagitis; in these six patients, the median maximum esophageal dose was 67.64 Gy (range, 38.34-74.40). Acute grade 2 esophagitis was associated with the maximum dose to the esophagus (OR 1.07, 95% CI 1.02-1.16, p=0.03) and occurred in 9% of patients with a maximum esophageal dose ≤60 Gy and 44% of those with doses >60 Gy (p=0.02). Grade 2 esophagitis was also not associated with ultracentral primary and/or node-positive disease (p=0.66). No patients developed late esophageal toxicity or esophageal toxicity requiring intervention such as enteral feeding tube or endoscopic dilation.

**Table 3:** Treatment Toxicity by Dose to Organs at Risk CTCAE, Common Terminology Criteria for Adverse Events; n, number, Gy, Gray. No Grade 4-5 adverse events were observed.

	CTCAE Version 4.0 Toxicity Grade				
	0	1	2	3	p-value
Pneumonitis, n (%)	26 (50)	17 (33)	7 (13)	2 (4)	-
Bilateral lung dose (%), median (range)					
5 Gy	45 (16-75)	42 (35-73)	45 (35-56)	43 (35-46)	0.98
20 Gy	20 (4-35)	22 (14-33)	22 (14-25)	24 (15-33)	0.35
30 Gy	15 (2-29)	15 (8-25)	18 (11-28)	19 (10-28)	0.15
40 Gy	10 (1-21)	10 (4-19)	14 (8-21)	16 (6-25)	0.27
Mean Lung Dose (Gy), median (range)	11.9 (3.4-17.9)	11.8 (7.7-20.3)	12.7 (8.4-19.1)	14.4 (8.2-20.5)	0.29
Esophagitis, n (%)	36 (69)	8 (15)	8 (15)	0 (0)	-
Esophagus Maximum Dose (Gy), median (range)	29.7 (11.6-75.1)	14.6 (4.7-65.0)	67.6 (38.3-74.4)	-	0.20

## Discussion

While surgery and SBRT are accepted therapeutic options for early stage NSCLC, the role of HRT alone in patients with more advanced disease or in those who are ineligible for chemotherapy, surgery, or SBRT is unclear. The results of CALGB 33904 demonstrated that HRT is a safe and effective option for nonsurgical stage I NSCLC with tumors ≤4 cm, but data are scarce regarding patients that do not meet these criteria [9]. Our data not only confirm the tolerability and reasonable efficacy of HRT in early stage disease, but also illustrate promising outcomes for tumors unsuitable for SBRT and in patients with limited nodal disease unable to receive chemotherapy.

The median OS for the cohort of stage I-IIB patients was 39.6 months-similar to the 38.5 months reported from the CALGB study for stage IA/IB patients. Among stage I patients, the three-year LF was 25%, which is less than what would be expected after SBRT [1-2,23], but higher than other HRT regimens described in the literature [17-18]. While multiple studies have noted improved tumor control with a BED10 >100 Gy using hypofractionated regimens, variation in statistical methods, dose prescription and delivery, treatment schedule, and definitions of recurrence limit the generalizability of this conclusion. In this analysis, the cumulative incidence of distant failure was 34% overall. This estimate was numerically, but not statistically significantly, higher for stage I patients compared to stage II patients. This may be due to the limited sample size. It may also be due to a higher competing risk of death in stage II patients, as more stage I patients would survive to have a local and/or distant failure event. This may also be reflective of the propensity for subsequent neoplasms in patients with a heavy smoking history. All patients in this cohort were either current or former smokers and the median smoking history was 50 pack-years, and the most common site of distant failure was the lung parenchyma for both groups.

Other recent studies have assessed the toxicity of SBRT with regard to central and ultracentral tumors [5,8,24]. In a series reviewing 65 patients with ultracentral tumors treated with SBRT in ≤8 fractions, Grade 3+ toxicity occurred in 19%, including 7 (11%) patients who experienced grade 5 toxicity [24]. In comparison, our reported fractionation was well tolerated: severe (CTCAE Grade 3+) toxicity was infrequent in this cohort primarily composed of patients with ultracentral or node-positive disease. Conclusions regarding lung dosimetric predictors were limited due to the low number of pneumonitis events. With regard to esophagitis, attempts should be made to limit the maximum dose to less than 60 Gy based upon our observation. However, in the node-positive and ultracentral population, this may not be feasible. In the RTOG 0617 phase III clinical trial, the primary esophageal dose constraint was a mean dose of <34 Gy. The maximum dose to the esophagus was recorded per protocol, but to our knowledge, analyses of esophagitis by maximum esophageal dose have not been reported. By comparison, in a prospective, phase I dose-escalation study of accelerated HRT to 60 Gy with concurrent chemotherapy, the esophagus was constrained to a maximum less than 105% of the prescription dose (63 Gy) and the proportion of the esophageal volume receiving >55 Gy was constrained to <30% [14]. In this report, our data support the assertion that HRT to 70.2 Gy is a safe and effective alternative with a reasonably low risk of grade 1-2 esophagitis.

One potential limitation to this regimen, in comparison with other regimens utilized for NSCLC not suitable for SBRT, is the patient inconvenience associated with 26 daily fractions. A Canadian, multi-institutional phase II trial included patients with T1-3N0 NSCLC that were ineligible for surgical resection due to underlying comorbidity or declined surgery [25]. HRT was delivered to a dose of 60 Gy in 15 fractions (BED10 = 84 Gy) using 3D-conformal radiotherapy (3D-CRT), resulting in favorable primary tumor control (87% at two years) and low rates of toxicity (10% grade 3-4 pneumonitis). Of note, this study excluded patients with hilar nodal involvement, primary tumors greater than 5 cm, centrally located T3 tumors, and patients with poor performance status (ECOG >2). In the current series, 21% of patients had N1 disease, nine of 52 had primary tumors >5 cm, and 15% had an ECOG performance status of 3, and yet the local control was comparable and the observed toxicity rates were slightly lower. This may be related to the difficulty of toxicity assessment via retrospective chart review, which likely underestimates these rates.

Although previous studies have described regimens that appear safe and efficacious for centrally-located, node-negative disease [6,26], data for HRT alone in the management of stage IIB disease with hilar involvement are lacking. A dose-escalation study assigned patients with stage II-IV NSCLC, poor performance status (ECOG 2 or greater) who were not candidates for resection, SBRT, or concurrent chemoradiotherapy to 50, 55, or 60 Gy in 15 fractions [27]. None of the dose levels exceeded the maximum tolerated dose and there were few grade ≥3 toxicities. No differences in survival were noted between groups, and local failure data were not reported due to poor survival. Dose-escalation to this extent (maximum dose level BED10 = 84 Gy) as reported in this study requires highly conformal techniques such as intensity modulated radiotherapy; the majority (90%) of patients in this study were treated using 3D conformal techniques without an increase in treatment toxicity. Additionally, the current series is comprised of patients with node-negative or limited (hilar) nodal disease with better performance status. In these patients, the risk of locoregional failure may outweigh the competing risk of death from other causes, warranting attempts to achieve a dose adequate for durable tumor control. A retrospective analysis [28] of stage II-III patients treated with HRT alone (93% to a dose of 66 Gy in 30 fractions, BED10 = 80.52 Gy) reported comparable survival in stage II patients (median OS 24 months), but two patients could not tolerate completion of RT and 59% experienced grade 2 esophagitis. No patients in the current series failed to complete the prescribed course of radiotherapy. It is our position that this fractionation scheme represents an acceptable mildly hypofractionated alternative for hilar node-positive patients that may be delivered with routine 3D-CRT. While SBRT or similar hypofractionated regimens may be appropriate for patients with node-negative, central and/or large NSCLC [29], a more protracted regimen may be appropriate for cases where normal tissue constraints are unable to be met using such fractionation. In these cases, in addition to those with hilar involvement, 70.2 Gy in 26 fractions appears efficacious and safe.

The addition of consolidative immunotherapy has recently been shown to improve survival after chemoradiotherapy in stage III NSCLC [30]. Investigations of the role for immunotherapy after definitive SBRT for early stage NSCLC are also planned. In light of these advances, immunotherapy after definitive HRT, as is being studied in similar cohorts after surgical resection (NCT02504372, NCT02273375), may further improve outcomes.

This study is limited by its retrospective nature with the inherent selection biases therein. In addition, limitations exist regarding the detection of toxicity events by review of the electronic medical record, and there exists a small risk of underestimation of these toxicity rates. The study is limited by its small sample size, reducing the power of statistical comparisons within this group. The long duration of the study period may also be problematic with regard to changes in practice patterns in the use of SBRT and HRT for central/ultracentral NSCLC that have shifted over time. As a result, these conclusions must be limited to hypothesis generation. It should be noted that treatment options are limited for this patient population not eligible for other standard of care therapies due to centrally-located disease, inoperable and/or unresectable disease or medical comorbidity precluding SBRT or surgery. Further study into similarly hypofractionated regimens is warranted to improve local control and reduce treatment time.

## Conclusions

Hypofractionated radiotherapy alone to 70.2 Gy is a safe and efficacious alternative for patients with stage I-IIB NSCLC who are ineligible for other standard of care interventions such as surgical resection or SBRT. This regimen results in good locoregional control, comparable survival outcomes, and an acceptable toxicity profile. Further prospective study is warranted.
